# Prognostic significance of squamous differentiation in pT2N0 urothelial bladder cancer

**DOI:** 10.3892/ol.2026.15654

**Published:** 2026-05-18

**Authors:** Alper Coskun, Ahmet Bilgehan Sahin, Selva Kabul, Mehmet Emin Dagtekin, Seda Sali, Buket Erkan Ozmarasali, Adem Deligonul, Erdem Cubukcu, Meral Kurt, Gursel Savci, Turkkan Evrensel, Ismet Yavascaoglu

**Affiliations:** 1 Department of Medical Oncology, Faculty of Medicine, Uludag University, 16059 Bursa, Turkey; 2 Department of Pathology, Faculty of Medicine, Uludag University, 16059 Bursa, Turkey; 3 Department of Internal Medicine, Faculty of Medicine, Uludag University, 16059 Bursa, Turkey; 4 Department of Radiation Oncology, Faculty of Medicine, Uludag University, 16059 Bursa, Turkey; 5 Department of Radiology, Faculty of Medicine, Uludag University, 16059 Bursa, Turkey; 6 Department of Urology, Faculty of Medicine, Uludag University, 16059 Bursa, Turkey

**Keywords:** bladder, cancer, squamous differentiation, survival, urothelial

## Abstract

Bladder cancer is the ninth most common malignancy worldwide, with urothelial carcinoma (UC) representing the predominant histopathological subtype. UC may present as pure urothelial carcinoma (PUC), or alongside other variant histologies (VHs), with squamous differentiation in UC (SD) being the most frequently observed variant. The present study provides a population-based insight into the survival outcomes of patients with SD compared with PUC in early-stage disease. The present retrospective, observational study analyzed pathology reports from all cystectomies (n=632) performed at Uludag University Faculty of Medicine (Bursa, Turkey) between December 2010 and December 2023. A total of 23 patients with SD and 45 patients with PUC were evaluated; all of the patients had pathological stage T2N0 (pT2N0). The median follow-up period was 5.27 years (range, 0.23–16.60). Kaplan-Meier survival analysis demonstrated no statistically significant difference in disease-free survival (DFS) or overall survival (OS) between the SD and PUC groups. Univariate Cox regression analysis identified that age at diagnosis was a potential prognostic factor of both DFS and OS. The findings suggest that SD does not significantly affect DFS or OS in patients with pT2N0 UC. However, further prospective, multicenter, large-scale studies are warranted to validate and expand upon these findings.

## Introduction

Bladder cancer (BC) is the ninth most common cancer worldwide (1). According to global data from 2022, ~615,000 new cases were diagnosed, and >220,000 deaths occurred due to BC, with the incidence notably higher in men than in women (2). Based on data from Cancer Statistics 2025, BC ranks as the fourth most common cancer in men and eighth in cancer-related deaths in the United States (US) (3). The median age at diagnosis is 69 years in men and 71 years in women (4,5). Although several risk factors for BC have been identified, cigarette smoking remains the most notable, particularly in Western populations (6). Tobacco smoke contains numerous carcinogens, including polycyclic aromatic hydrocarbons and N-nitrosamines, which are implicated in bladder carcinogenesis (7).

From a histopathological perspective, urothelial carcinoma (UC) is the predominant subtype of BC, accounting for ~90% of cases in the US and Western Europe. By contrast, non-urothelial bladder cancer (NUC) is more prevalent in regions such as the Middle East, largely due to the endemic presence of schistosomiasis, an established risk factor (8). In a retrospective analysis of 72,452 patients with UC and NUC in the US, it was found that adenocarcinoma was diagnosed at younger ages, that non-squamous NUCs were more common in males, and that squamous cell carcinoma (SqCC) and small cell neuroendocrine carcinoma subtypes were associated with poorer prognoses than UC. The same study reported that >60% of NUC cases were diagnosed at advanced stages, while nearly 80% of UC cases were identified at early stages (9).

UC may present as pure urothelial carcinoma (PUC) or with variant histologies (VHs), including squamous, glandular, micropapillary, trophoblastic, sarcomatoid, nested and neuroendocrine components. Among these, squamous differentiation in UC (SD) is the most common, found in ~20% of UC cases (10,11). SD is characterized histologically by intercellular bridging and keratinization (12). In one study, the incidence of high-stage disease (T3-T4) in patients with SD was 72.3%, compared with 43.1% in patients with PUC (11). Furthermore, nodal involvement was more frequent in patients with diffuse SD (46.2%) than in those with PUC (27%). Certain studies have suggested that SD is associated with a more aggressive clinical course and poorer response to adjuvant chemotherapy (CT) and radiotherapy (RT) compared with PUC (13–15). However, evidence on the prognostic impact of SD remains controversial. For example, Wasco *et al* (16) reported that although VHs, including SD, were associated with higher T stages and more frequent muscle invasion or extravesical extension, they did not significantly affect disease-free survival (DFS). By contrast, another study found that the presence of VHs in UC was associated with worse survival outcomes, particularly with micropapillary, plasmacytoid and small cell variants, while SD demonstrated similar survival to PUC (17). Although SD has been associated with advanced stage and poor prognosis in some studies, its impact in pathological stage T2N0 (pT2N0) UC remains unclear (18–26).

The present study aimed to evaluate the clinical features, treatment response and survival outcomes of patients with pT2N0 UC exhibiting squamous differentiation in comparison with those with PUC. It is hypothesized that the study will contribute to an improved understanding of the clinical course and treatment strategies of pT2N0 UC with SD.

## Materials and methods

### Study population

The present study is a clinical, observational, retrospective cohort study. Pathology reports of all cystectomy procedures (n=632) performed at Uludag University Faculty of Medicine (Bursa, Turkey) between December 2010 and December 2023 were reviewed retrospectively. Demographic and clinical data were extracted from 115 patients with either SD or PUC and a pathological stage of pT2N0 at cystectomy. The data were obtained from the institutional hospital automation system. This study was approved by the Uludag University Faculty of Medicine Clinical Research Ethics Committee (Bursa, Turkey; approval no. 2025/4-26). The inclusion criteria were as follows: i) Patients aged ≥18 years; ii) histopathological diagnosis of UC with or without SD; iii) pathological stage pT2N0 at the time of cystectomy; iv) no evidence of distant metastasis and v) availability of complete clinical and follow-up data. The exclusion criteria were as follows: i) <18 years of age; ii) histological differentiation other than SD; iii) distant metastases at diagnosis; and iv) did not undergo curative surgery.

### Data collection

The data were obtained through retrospective review of hospital electronic records and archived clinical files. The following variables were recorded: i) Demographic, clinical and pathological characteristics; ii) smoking history; iii) comorbidities; iv) stage at diagnosis; v) the Bacillus Calmette-Guerin treatment; vi) surgical procedures; vii) adjuvant systemic treatments (CT and/or RT); viii) recurrence status; ix) survival status; and x) last follow-up date.

### Histopathological examination

**Figure 1.**
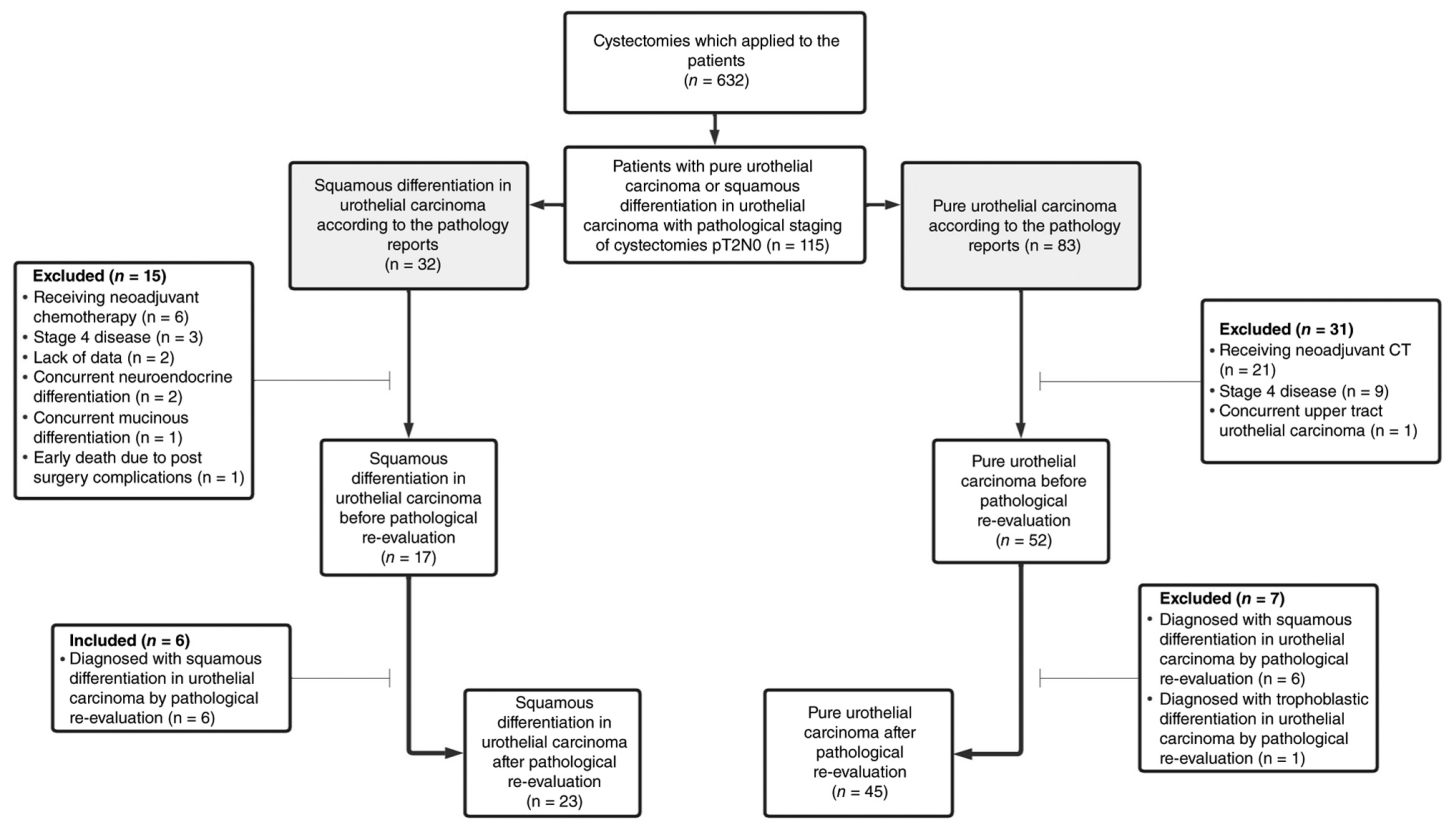
Study flow diagram of patient selection and histopathological re-evaluation. A total of 632 patients who underwent cystectomy were screened, of whom 115 patients with pT2N0 UC were identified based on initial pathology reports. After applying predefined exclusion criteria (including neoadjuvant CT, stage IV disease, missing data, concurrent VHs and early postoperative mortality), 69 patients underwent histopathological re-evaluation. Following re-evaluation, 23 patients were classified as having SD and 45 as PUC; one patient with a non-squamous VH was excluded. pT2N0, pathological stage T2N0; UC, urothelial carcinoma; VH, variant histology; SD, squamous differentiation in urothelial carcinoma; PUC, pure urothelial carcinoma.

Histopathological evaluation was performed on formalin-fixed, paraffin-embedded tissue sections using routine hematoxylin and eosin staining. Tissue samples were fixed in 10% neutral buffered formalin at room temperature for 24–48 h. Paraffin-embedded sections were cut at a thickness of 4 µm. Hematoxylin and eosin staining was performed at room temperature, with hematoxylin staining for 5 min followed by eosin staining for 2 min. All slides were reviewed by an experienced genitourinary pathologist according to the 2022 World Health Organization classification criteria (27) using light microscopy. Histopathological re-evaluation was performed in a subset of patients (n=69) after the application of the predefined exclusion criteria. Based on the initial pathology reports, 15 patients with SD and 31 patients with PUC were excluded (Fig. 1). The remaining 69 patients underwent review by a genitourinary pathologist. Among these, six patients initially reported as PUC were reclassified as squamous differentiation and subsequently included in the SD group. In addition, one patient with a non-squamous VH (trophoblastic differentiation) was excluded. Only patients with confirmed pT2N0 disease after pathological re-evaluation were included in the final analysis. The study flow diagram illustrating the patient selection process is presented in Fig. 1.

### Statistical analysis

Statistical analyses were conducted using IBM SPSS Statistics version 25.0 (IBM Corp.). Categorical variables were summarized as frequencies and percentages, and continuous variables were expressed as the mean ± standard deviation, median and range (minimum-maximum). Kolmogorov-Smirnov and Shapiro-Wilk tests were used to assess normality of distribution. Comparisons between two independent groups were performed using the independent samples t-test for normally distributed variables and the Mann-Whitney U test for non-normally distributed variables. Survival analyses were performed using the Kaplan-Meier method, and survival curves were generated accordingly. DFS was defined as the time from curative surgery to recurrence, last follow-up or death. Overall survival (OS) was defined as the time from curative surgery to death or last follow-up. Univariate Cox regression analysis was used to identify factors associated with survival outcomes. P<0.05 was considered to indicate a statistically significant difference.

## Results

The median age of the patients was 66 years (range, 43–80 years), the majority were men and the median follow-up duration was 5.27 years (range, 0.23–16.60 years). The most common comorbidity was hypertension, which was present in 37 patients (54.4%). Among the 68 patients included in the present study, the SD group consisted of 23 patients. Comparative analysis revealed no significant differences between the two groups in terms of demographic and clinical characteristics, except for sex. Adjuvant CT was administered to only four patients (two in each group). Given the very limited number of cases, no meaningful comparison or subgroup analysis could be performed. Baseline characteristics of the study population are summarized in Table I.

**Figure 2.**
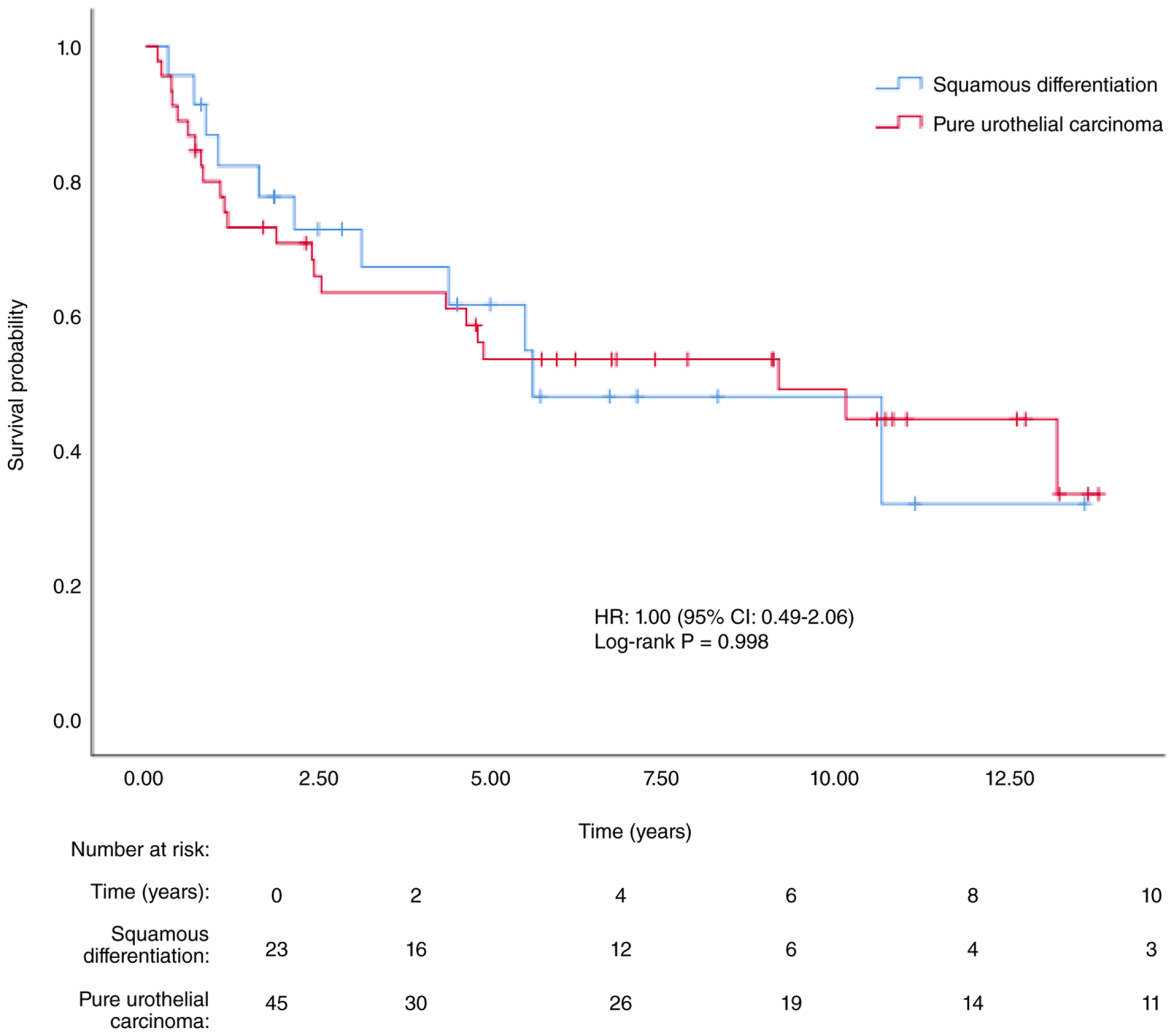
Kaplan-Meier curve for disease-free survival according to histopathological subtype.(SD vs. PUC; log-rank P=0.998). SD, squamous differentiation in urothelial carcinoma; PUC, pure urothelial carcinoma; HR, hazard ratio; CI, confidence interval.

**Figure 3.**
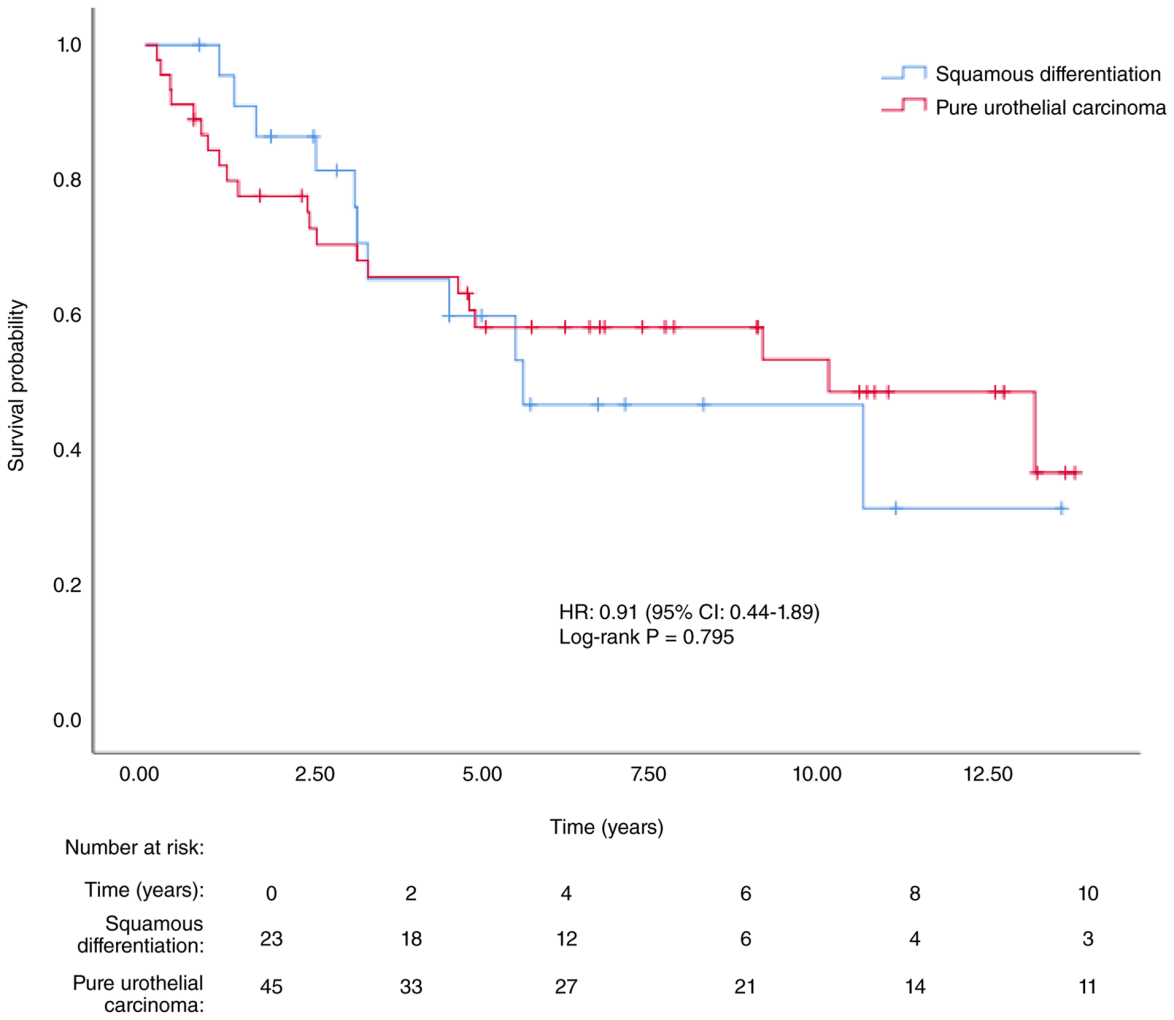
Kaplan-Meier curve for overall survival according to histopathological subtype (SD vs. PUC; log-rank P=0.795). SD, squamous differentiation in urothelial carcinoma; PUC, pure urothelial carcinoma; HR, hazard ratio; CI, confidence interval.

Recurrence occurred in seven patients (30.4%) in the SD group and 10 patients (22.2%) in the PUC group. A total of 11 patients (47.8%) in the SD group and 21 patients (46.7%) in the PUC group died. The median DFS was 5.61 years [95% confidence interval (CI), 0.63–10.59] in the SD group and 9.17 years (95% CI, 1.64–16.70) in the PUC group (P=0.998). The median OS was 5.61 years (95% CI, 0.58–10.64) in the SD group and 10.15 years (95% CI, 3.49–16.82) in the PUC group (P=0.795). Kaplan-Meier survival curves are presented in Figs. 2 and 3.

Univariate Cox regression analysis was performed to identify potential prognostic factors associated with survival outcomes. In the univariate Cox regression analysis, age at diagnosis emerged as a potential prognostic factor of both DFS and OS. The detailed results of the univariate Cox regression analysis for DFS and OS are presented in Table II.

## Discussion

In the present study, no significant differences in survival were observed between patients with SD and those with PUC. This finding was observed in a highly selected cohort of patients who did not receive neoadjuvant therapy, had no distant metastasis and had pT2N0 disease.

Transcriptomic profiling studies have demonstrated that BC can be classified into molecular subtypes, including luminal and basal-like tumors (28). Basal tumors are more frequently associated with squamous features and tend to have a more aggressive clinical course, whereas luminal tumors are linked to a more favorable prognosis and distinct therapeutic targets (28,29). It has also been reported that basal subtype tumors may have an improved response to immune checkpoint inhibitors (ICIs), while luminal tumors may benefit more from fibroblast growth factor receptor 3- and human epidermal growth factor receptor 2-targeted therapies (30). Moreover, SqCC has been associated with responsiveness to epidermal growth factor receptor-targeted agents (29).

Within this biological framework, the clinical importance of squamous differentiation remains to be clarified. In this context, it is important to distinguish SD from SqCC, which is defined by complete squamous differentiation (30). SD is considered an intermediate phenotype between SqCC and UC based on immunohistochemical characteristics (31). While treatment responses and prognostic implications have been relatively well described for SqCC, data on the clinicopathological features and survival outcomes of patients with SD remain limited, particularly in early-stage disease.

The patient population in the present study represents a highly specific and distinct cohort. All patients included had a diagnosis of either SD or PUC, a pathological stage of T2, no lymph node involvement or distant metastasis, had not received neoadjuvant systemic therapy, and underwent cystectomy. To the best of our knowledge, no prior study in the literature has examined a cohort with such narrowly defined inclusion criteria. The majority of studies in the literature involve broader patient populations, including varying pathological stages and the presence of nodal or distant metastases, which may explain discrepancies in reported outcomes. For example, Minato *et al* (18) evaluated clinical characteristics and survival outcomes in 101 patients with cT2-T4aN0M0 disease who underwent radical cystectomy (PUC, n=81; SD, n=20). Unlike the present study, the multivariate analysis identified the presence of squamous differentiation as a factor negatively affecting OS. However, the cohort included a higher proportion of advanced-stage patients, with 60% of the SD group and 30.9% of the PUC group diagnosed with pathological stage T3 or higher, and nodal positivity reported in 45 and 21% of cases, respectively. Notably, only eight patients in the SD group had stage ≤pT2 disease (18). Izard *et al* (19) conducted another large study including 178 patients with SqCC, 325 with SD and 2,884 with PUC. Similar to the present study, a higher proportion of female patients was noted in the SD group. However, in contrast to the findings of the present study, their cohort included ~40% of patients with T3 stage disease and 25% with lymph node positivity. Furthermore, T3 stage disease was more common in the SD and SqCC groups, while T1 disease was more prevalent in the PUC group. The 5-year OS rates were reported as 34% for SD and 28% for PUC. In their multivariate analysis, factors that were independently associated with OS included age ≥70 years, advanced T and N stages and positive surgical margins. In the present univariate analysis, age at diagnosis was associated with both DFS and OS. Other variables could not be compared due to the homogeneity of the present cohort, whereby all patients had pT2N0 disease with negative surgical margins (19). Several studies have reported findings consistent with the present study, demonstrating no notable survival difference between SD and PUC following cystectomy (20–24). By contrast, certain studies have suggested a negative prognostic impact of squamous differentiation, particularly in cohorts including more advanced-stage disease (18,25). These discrepancies may largely be explained by differences in patient selection, particularly with respect to pathological stage and nodal involvement.

Previous studies have suggested that patients with progressive T2 disease following non-muscle-invasive bladder cancer may have worse oncological outcomes compared with those with *de novo* disease (32–34). By contrast, the present study did not demonstrate a significant difference between these groups, although this finding should be interpreted with caution due to the limited sample size.

In a study evaluating cancer-specific survival (CSS) and OS in 117,275 patients with BC using the Surveillance, Epidemiology, and End Results database from 2004 to 2015, age ≥75 years at diagnosis was identified as an independent factor associated with poorer CSS and OS outcomes (35). Similarly, the present study demonstrated that age ≥65 years at the time of diagnosis was a potential risk factor negatively impacting both DFS and OS.

In the study conducted by Laymon *et al* (36) the clinicopathological characteristics and survival outcomes of 223 patients with SD and 318 patients with SqCC were evaluated. The findings showed that lymph node involvement and extravesical extension were more common among patients with SD, and the 5-year recurrence-free survival was significantly worse in the SD group compared with the SqCC group (36). By contrast, in the present study a comparative analysis between patients with SD and those with PUC was conducted, allowing specific examination of the prognostic implications of squamous differentiation within the context of UC.

Smoking is a well-established risk factor for BC and has been associated with more aggressive tumor biology and VHs, including SD (37,38). In the present study, smoking status was comparable between groups and was not associated with survival outcomes, possibly due to the limited sample size.

Notably, a significant difference in sex distribution was observed between the SD and PUC groups in the present study. Although definitive evidence is lacking, previous studies have suggested that VHs, including SD and SqCC, may be relatively more frequent in women compared with PUC, potentially due to differences in carcinogen exposure, hormonal factors and delays in diagnosis (39,40). The findings of the present study appear to be consistent with these observations. However, this result should be interpreted with caution given the relatively small sample size and the retrospective design of the study.

The present study had several limitations. The most notable is its single-center, retrospective design and relatively small sample size, particularly in the SD group, which may have limited the statistical power to detect significant differences, thereby increasing the risk of a type II error. Additionally, only four patients received adjuvant CT, and none received ICIs, limiting the ability to evaluate the impact of adjuvant treatment on survival outcomes. The exclusion of patients who received neoadjuvant CT further restricted the generalizability of the findings. Another important limitation is the lack of quantitative assessment of the extent of squamous differentiation. Although histopathological re-evaluation was performed by a dedicated genitourinary pathologist, the available specimens and pathology reports did not include a standardized or reproducible quantification of the squamous component. Therefore, the prognostic impact of the percentage of squamous differentiation was not reliably assessed. Future prospective studies with standardized pathological reporting are needed to clarify this issue. Finally, multivariate Cox regression analysis was not performed due to the limited number of events. Therefore, only univariate analysis was considered appropriate.

In conclusion, the present study demonstrated that the presence of squamous differentiation does not impact DFS or OS in patients with pT2N0 UC who underwent cystectomy without prior neoadjuvant treatment or evidence of metastasis. Although existing literature presents conflicting results, the findings align with the majority of studies suggesting no prognostic difference between SD and PUC in this patient population. However, these findings should be interpreted with caution and may not be generalizable to patients with more advanced pathological stages, nodal involvement or those receiving neoadjuvant systemic therapy. Larger, prospective, multi-center studies are warranted to establish more definitive conclusions.

## Figures and Tables

**Table I.** Baseline clinicopathological characteristics of the study population.

Characteristics (n=68)	Squamous differentiation (n=23)	Pure urothelial carcinoma (n=45)	P-value
Age, median (range)	67 (48–77)	65 (43–80)	0.183
Sex (%)			0.028
Male	18 (78.3)	43 (95.6)	
Female	5 (21.7)	2 (4.4)	
Comorbidity (%)			
Hypertension	11 (47.8)	26 (57.8)	0.439
Diabetes mellitus	4 (17.4)	7 (15.6)	0.847
IHD	3 (13.0)	14 (31.1)	0.106
Asthma/COPD	3 (13.0)	12 (26.7)	0.203
Smoking status^a^, n (%)			0.191
Yes	7 (30.4)	19 (42.2)	
No	5 (21.7)	3 (6.7)	
Missing	11 (47.8)	23 (51.1)	
Smoking (pack/year), mean ± SD	39.0±6.7	39.8±3.7	0.918
*De novo* pT2, n (%)	15 (65.2)	34 (75.6)	0.372
BCG, n (%)	2 (8.7)	6 (13.3)	0.577
Type of surgery, n (%)			0.377
RCP	18 (78.3)	39 (86.7)	
RC	5 (21.7)	6 (13.3)	
Adjuvant chemotherapy, n (%)	2 (8.7)	2 (4.4)	0.484
Follow-up duration, years mean ± SD	5.76±0.75	6.49±0.68	0.697

a Percentages are calculated based on available data (excluding missing values). BCG, Bacillus Calmette-Guérin; COPD, chronic obstructive pulmonary disease; IHD, ischemic heart disease; RC, radical cystectomy; RCP, radical cystoprostatectomy.

**Table II.** Univariate cox regression analysis of factors associated with DFS and OS.

A, DFS				

		95% CI		
			
Variable	HR	Lower	Upper	P-value
Age^a^	1.089	1.029	1.152	0.003
Sex^b^	0.228	0.031	1.667	0.145
Smoking status^c^	2.325	0.517	10.448	0.271
Smoking (pack/year)	1.000	0.966	1.035	0.994
Pathological subtype^d^	0.999	0.486	2.057	0.998
*De novo* pT2^e^	0.558	0.274	1.139	0.109
BCG^f^	0.631	0.192	2.069	0.447
Adjuvant CT^g^	0.305	0.042	2.238	0.243
B, OS				

		**95% CI**		
			
**Variable**	**HR**	**Lower**	**Upper**	**P-value**

Age^a^	1.099	1.036	1.167	0.002
Sex^b^	0.251	0.034	1.840	0.174
Smoking status^c^	1.970	0.429	9.038	0.383
Smoking (pack/year)	0.998	0.961	1.037	0.920
Pathological subtype^d^	0.908	0.436	1.891	0.796
*De novo* pT2^e^	0.519	0.251	1.074	0.077
BCG^f^	0.702	0.213	2.311	0.560
Adjuvant CT^g^	0.326	0.044	2.398	0.271

Reference groups:

a Age <65;

b male sex;

c no smoking;

d squamous differentiation in urothelial carcinoma,

e Ta/T1 disease at diagnosis;

f no BCG;

g no CT. BCG, Bacillus Calmette-Guérin; CI, confidence interval; CT, chemotherapy; HR, hazard ratio; DFS, disease-free survival; OS, overall survival.

## Data Availability

The data generated in the present study may be requested from the corresponding author.
